# Kinematic and kinetic comparison between preprofessional pitchers from the Dominican Republic and the United States

**DOI:** 10.3389/fspor.2023.1152474

**Published:** 2023-04-18

**Authors:** Kristen F. Nicholson, Joseph A. Mylott, Tessa C. Hulburt, Tyler J. Hamer, Garrett S. Bullock

**Affiliations:** ^1^ Department of Orthopaedic Surgery, Wake Forest School of Medicine, Winston Salem, NC, United States; ^2^ Department of Biomechanics, University of Nebraska at Omaha, Omaha, NE, United States; ^3^ Department of Biostatistics and Data Science, Wake Forest School of Medicine, Winston Salem, NC, United States; ^4^ Centre for Sport, Exercise and Osteoarthritis Research Versus Arthritis, University of Oxford, Oxford, United Kingdom

**Keywords:** throwing, overhead athlete, biomechanics, shoulder, elbow

## Abstract

**Introduction:**

Pitching biomechanical efficiency is defined as the association between pitch velocity and arm kinetics. Pitching mechanics inefficiency, an increase in arm kinetics without the resultant increase in pitch velocity, can lead to increased arm strain, increasing arm injury risk. The purpose of this study was to compare arm kinetics, elbow varus torque and shoulder force, in preprofessional United States (US) and Dominican Republic (DR) pitchers. Kinematics that are known to influence elbow varus torque and shoulder force as well as a representative of pitch velocity (hand velocity) were also compared.

**Methods:**

A retrospective review was performed on baseball pitchers from the DR and US who participated in biomechanical evaluations conducted by the University biomechanics laboratory personnel. Three-dimensional biomechanical analyses were performed on US (*n* = 37) and DR (*n* = 37) baseball pitchers. Potential differences between US and DR pitchers were assessed through analysis of covariance with 95% confidence intervals [95% confidence Interval (CI)].

**Results:**

Preprofessional DR pitchers experienced increased elbow varus torque compared with their US counterparts [DR: 7.5 (1.1); US: 5.9 (1.1) %BWxH; Beta: −2.0 (95% CI: −2.7, −1.2) %BWxH], despite throwing fastballs with slower hand velocity [DR: 3,967.1 (939.4); US: 5,109.1 (613.8) °/s; Beta: 1,129.5 (95% CI: 677.5, 1,581.4) °/s]. DR and US pitchers demonstrated similar shoulder force [DR: 136.8 (23.8); US: 155.0 (25.7); Beta: 0.4 (95% CI: −1.2, 19.7) %BW].

**Discussion:**

Increased elbow varus torque although decreased hand velocity suggests inefficient pitching mechanics among DR pitchers. Inefficient pitching mechanics and increased elbow torque should be considered when developing training programs and pitching plans for professional pitchers from the Dominican Republic.

## Introduction

Musculoskeletal injuries in baseball athletes are a persistent and significant problem, with the greatest incidence attributed to shoulder and elbow injuries (1, 2). Overuse conditions and throw-related injuries are common, and arm injury incidence continues to increase (3). Pitching arm injuries are often attributed to excessive shoulder force and elbow varus torque (4, 5). The rapid acceleration and deceleration of the arm during pitching create high forces within the glenohumeral joint, leading to increased humeral distraction that is resisted by several static and dynamic stabilizers (5, 6). This excessive shoulder force required to resist distraction can result in symptomatic rotator cuff injury, labral pathology, and other injuries to the shoulder (5, 6). In addition to shoulder stress, pitching places high levels of stress on the medial elbow, where torque can exceed 110 Nm, during the late cocking phase (7, 8). These high torques, coupled with the repetitive exposure to overhead throwing, are significant contributors to the ulnar collateral ligament (UCL) tears and elbow valgus overload syndrome (9).

Pitching biomechanical efficiency is defined as the association between pitch velocity and arm kinetics, such as shoulder force and elbow varus torque (10). Efficient pitching mechanics allow for maximized pitch velocity while minimizing arm kinetics. Pitching inefficiency can increase arm strain and injury risk (11). Recent literature has shown that pitch velocity and kinematic variables of maximum humeral rotation velocity, shoulder abduction at foot strike, and maximum shoulder external rotation significantly influence both elbow varus torque and shoulder force in baseball pitchers from the United States (US) (11). In addition, maximum humeral rotation velocity, among other kinematic variables, has a significant influence on pitch velocity (12). When considered together, these variables can help us begin to understand concepts, such as pitching efficiency and ideal pitching mechanics. As such, understanding potential inefficiencies in pitching kinematics and kinetics can elucidate shoulder and elbow injury risk.

Training habits and playing exposure prior to signing a professional baseball contract may impact pitching mechanical efficiency and predispose baseball pitchers to different injury risks (13, 14). A majority of previous pitching biomechanics studies have analyzed American or Japanese cohorts. However, Major League Baseball (MLB) continues to increase worldwide participation, with over 25% of all MLB players coming from outside North America (15, 16). The largest population of MLB foreign-born players reside in the Dominican Republic (DR), at a prevalence of 10% (16). In a sample of over 1,000 US and foreign-born amateur youth baseball pitchers, foreign-born pitchers threw an average of one to two standard deviations faster and farther than American pitchers (13). In another sample of over 130 US- and DR-born amateur baseball players that recently signed professional baseball contracts, DR-born players exhibited a greater prevalence of poor core control and deficient performance of fundamental movement patterns compared with the US-born players (14). While these previous studies highlight differences in pitching velocity and functional movement, differences in pitching efficiency and injury risk among pitchers from the US and DR pitchers are poorly understood. There is a need to quantify these potential differences as pitchers enter the MLB organization. Most pitchers start their professional baseball careers in developmental and minor league play. Understanding pitching strategies and injury risk at the time of entry could be invaluable during this developmental period.

Shoulder force and elbow varus torque are markers of arm stress among all pitchers; however; DR pitchers' mechanics and velocity-producing strategies may differ from their American counterparts, resulting in differences in throwing arm kinetics. Therefore, the purpose of this study is to compare elbow varus torque, shoulder force, and kinematics, that influence these arm stress variables in preprofessional pitchers from the US and the DR. We hypothesize that US and DR pitchers will have similar pitching strategies with no significant differences in kinetics or kinematics.

## Methods

A retrospective review was performed on baseball pitchers from the DR and US who participated in biomechanical evaluations conducted by the University biomechanics laboratory personnel. This study was approved by the University Institutional Review Board.

Three-dimensional biomechanical analyses were performed on US (*n* = 37) and DR (*n* = 37) baseball pitchers. Inclusion criteria included: (1) baseball players with a pitcher as their primary position; (2) being able to participate in all training, practices, and competitions; and (3) aged 16 and above. Exclusion criteria comprised: (1) signed a professional baseball contract at the time of data collection, (2) baseball players that played a position other than pitcher as their primary position within the same season, (3) shoulder or elbow injury within 6 months of the biomechanical evaluation. All US pitchers were collegiate-level pitchers on a National Collegiate Athletic Association Division 1 program. All DR pitchers were prospects for the upcoming MLB draft.

Data were examined from reports generated as part of a pitching evaluation. As part of the evaluation, 3D motion data were collected using the 40 retro-reflective markers set required for PitchTrak (17), and a 12-camera motion analysis system (Qualisys AB, Göteborg, Sweden). Motion data were collected at 400 Hz. Each pitcher went through a normal pregame warm-up period, before pitching four fastballs, four to eight breaking balls, and four changeups to a catcher receiving throws at a regulation distance (18.4 m). Fastballs were always thrown first, and only fastball data were analyzed for this study. Pitching models were defined using the PitchTrak model, and segment coordinate systems were defined according to ISB recommendations (17, 18). Data were processed and variables were calculated with Visual3D (C-Motion, Inc., Germantown, MD). Shoulder force was calculated as the maximum joint force between the upper arm and the trunk and normalized by body weight (*N*). Elbow varus torque was calculated as the maximum joint torque between the forearm and upper arm on the medial/lateral axis and was normalized by body weight multiplied by height (Nm). Variables of two or more fastballs were averaged for each pitcher. Maximum hand velocity was used as a representation of ball velocity. Hand velocity was defined as the angular velocity of the pitching hand segment in the laboratory's coordinate system in the direction of home plate. The maximum hand velocity occurred at ball release.

Elbow varus torque and shoulder force are influenced by player height, mass, age (19, 20), hand dominance (21), and pitch velocity (22). The torque and force values are normalized by body weight times height and body weight, respectively, to account for the influence of height and mass. Age, hand dominance, and pitch velocity are known to influence elbow torque and shoulder force; therefore, age, hand dominance, and hand velocity will be controlled within the analyses. Controlling confounding variables will give insight into how pitching mechanics influence pitching arm kinetics.

Missing data were assessed before analyses. Missing data were minimal (<1%) and complete case analyses were performed. Descriptive statistics were calculated for the mean (standard deviation) and count (percent). The potential difference between DR and US pitchers were assessed through analysis of covariance (ANCOVA) with 95% confidence intervals [95% confidence interval (CI)]. Sensitivity analyses were performed with raw elbow and shoulder kinetic data and included mass and height as covariates within the models. Further sensitivity analyses were performed to evaluate the potential influence of the random effects of each pitcher and repeated pitching measurements on the results. A series of mixed models, with a random effect at the individual pitcher level, were performed on both kinetic and kinematic data. All analyses were performed in R 4.1.2 R Core Team (2021). R: A language and environment for statistical computing. R Foundation for Statistical Computing, Vienna, Austria. URL https://www.R-project.org/, with the *glm* function used for model development.

## Results

Seventy-four pitchers were included in this study. United States pitchers demonstrated decreased elbow varus torque, increased shoulder force, decreased maximum shoulder external rotation, decreased maximum humeral rotation velocity, and increased maximum hand velocity (pitch velocity) when compared with DR pitchers (Table 1).

**Table 1:** Participant descriptives.

Variable	All participants (*n* = 74)	United States (*n* = 37)	Dominican Republic (*n* = 37)
Age (years)	19.2 (1.7)	20.1 (1.6)	18.2 (1.2)
Height (cm)	186.8 (6.3)	187.2 (7.1)	186.4 (5.6)
Weight (kg)	86.4 (11.2)	93.2 (10.7)	79.5 (6.4)
Body mass index (kg/m^2^)	24.7 (2.8)	26.6 (2.7)	22.9 (1.2)
Hand dominance (%Left)	28	35	22
Maximum elbow varus torque (%BWxH)	6.7 (1.3)	5.9 (1.1)	7.5 (1.1)
Maximum shoulder force (%BW)	145.9 (26.2)	155.0 (25.7)	136.8 (23.8)
Shoulder abduction at foot strike (°)	85.9 (10.8)	86.2 (10.6)	85.6 (11.1)
Maximum shoulder external rotation (°)	172.2 (12.7)	168.2 (11.9)	176.0 (12.5)
Maximum humeral rotation velocity (°/s)	5,545.9 (485.3)	5,423.3 (502.4)	5,668.5 (440.9)
Maximum hand velocity (°/s)	4,538.1 (975.4)	5,109.1 (613.8)	3,967.1 (939.4)

Results are reported as mean (standard deviation) or percentage for count data.

BW, body weight; H, height.

Statistical results before and after adjusting for confounding variables can be seen in Table 2. When controlling for confounding variables (i.e., holding age, hand dominance, and pitch velocity constant), United States pitchers demonstrated a further reduction in elbow varus torque compared with DR pitchers [Beta: −2.0 (95% CI: −2.7, −1.2) %BWxH, *p* < 0.001]. There were no differences in shoulder force between the United States and DR pitchers [0.4 (95% CI: −1.2, 19.7) %BW, *p* = 0.566]. Further, when controlling for age and hand dominance, United States pitchers maintained an increased hand velocity compared with DR pitchers [1,129.5 (95% CI: 677.5, 1,581.4) °/s, *p* < 0.001]. Sensitivity analyses results on non-normalized, raw elbow and shoulder kinetics data demonstrated similar results [elbow: −18.9 (95% CI: 029.9, −7.9) Nm, *p* = 0.001; shoulder: 137.3 (95% CI: −2.1, 276.7) N, *p* = 0.058]. Performing random effect models demonstrated similar kinetic results [elbow: −1.6 (95% CI: −2.2, −1.0) Nm; shoulder: 17.8 (95% CI: 9.9, 31.9) N]. Performing random effect models demonstrated similar kinematic results [maximum shoulder rotation velocity: −4.2 (95% CI: −10.9, 2.4) °/s; shoulder abduction at footstrike: −0.4 (95% CI: −6.6, 5.8) °; maximum humerus angular velocity: −190.7 (95% CI: −497.0, 115.9) °/s; maximum hand velocity: 1,105.9 (95% CI: 658.2, 1,552.7) °/s].

**Table 2:** Statistical comparison.

Variable	Unadjusted for confounders	Adjusted for confounders
Maximum elbow varus torque (%BWxH)	−1.5 (−2.0, 1.0)^*^	−2.0 (−2.7, −1.2)^*^
Maximum shoulder force (%BW)	18.2 (6.9, 29.4)^*^	0.4 (−1.2, 19.7)
Shoulder abduction at foot strike (°)	0.6 (−4.3, 5.6)	−0.7 (−6.9, 5.5)
Maximum shoulder external rotation (°)	−7.8 (−13.4, −2.3)^*^	−4.3 (−10.9, 2.9)
Maximum humeral rotation velocity (°/s)	−245.2 (−460.5, −29.8)^*^	−230.2 (−498.6, 38.1)
Maximum hand velocity (°/s)	1,142.1 (780.5, 1,503.6)^*^	1,129.5 (677.5, 1,581.4)^*^

Results are reported as the difference between United States pitchers and Dominican Republic pitchers (95% confidence interval).

Confounding variables include age, hand dominance, and hand velocity.

^*^ *p* < 0.05; BW, body weight; H, height.

## Discussion

The main findings of this study were contrary to our hypothesis of no significant differences. Preprofessional DR pitchers demonstrated increased elbow varus torque, maximum shoulder external rotation, maximum humeral rotation velocity, decreased shoulder force, and hand velocity compared with US pitchers. When controlling for age, handed dominance, and hand velocity, DR pitchers had even greater elbow varus torque when compared with their US counterparts, but the differences in shoulder force, maximum shoulder external rotation, and maximum humeral rotation velocity were eliminated. Before controlling for confounding variables, the US pitchers had higher shoulder force, which is expected with higher velocity pitches. The lack of a difference after controlling velocity is likely related to kinematics not being analyzed in this study. Trunk mechanics, sequencing, and shoulder abduction at release, have been found to significantly influence shoulder force, but not impact elbow torque (11, 12). DR pitchers may have appropriate trunk flexion, timing of rotations, and arm path mechanics that allow low shoulder force, while being inefficient in other kinematics that led to increased elbow stress.

Reducing elbow varus torque may be key to limiting UCL injuries in professional pitchers (23). Pitch velocity has been shown to be a primary contributor to elbow varus torque (11, 22). However, pitching mechanics have also been shown to influence elbow varus torque (11, 12), suggesting that there are ideal or efficient mechanics, that can help reduce pitching arm stress without sacrificing pitch velocity. Preprofessional DR pitchers throw fastballs with slower velocity while experiencing increased elbow varus torque compared with their US counterparts. This is contrary to previous research, (13) where DR pitchers threw faster than their US counterparts. However, this previous research included only younger adolescents, with US participants from summer baseball camps and DR participants from local baseball clubs. Among these cohorts, the DR pitchers were likely of higher caliber with more consistent coaching and skill development. This study sampled older professional pitching prospects, where US pitchers have better access to coaching and development, potentially inciting a reversal in throwing velocity trends. Ball velocity is the primary contributor to elbow varus torque, so, controlling hand velocity further increased the elbow varus torque discrepancy between US and DR preprofessional pitchers.

Maximum humeral rotation velocity and maximum shoulder external rotation have been shown to influence elbow torque (11) and ball velocity (24, 25). DR pitchers demonstrated increased humeral rotation velocity and maximum shoulder external rotation despite their decreased ball velocity. When controlling for age and hand dominance, significant differences in maximum shoulder external rotation and maximum humeral rotation velocity were eliminated. This is most likely due to the higher percentage of left-handed pitchers in the US cohort. Left-handed pitchers have decreased humeral torsion and less passive external rotation, limiting the time they must generate humeral rotation velocity (21, 26).

Higher elbow torque with slower pitching velocity suggests that DR pitchers utilize a less efficient pitching pattern. In addition, increased humeral rotation velocity and shoulder external rotation without a subsequent increase in pitching velocity indicates that DR pitchers may have limited ability to transfer energy through the kinetic chain. Preprofessional US pitchers have improved access to strength and conditioning resources and plans. Further, there are nutritional diet differences between countries, which may influence differences in body composition between players (27). This is reflected in the greater weight and BMI among US pitchers in the current study. Greater weight and BMI in an athletic population may indicate more strength. Greater strength may allow US pitchers to limit elbow torque by transferring energy more successfully (19, 20). Within cadaveric studies, the flexor-pronator mass supports and stabilizes the elbow during varus torque moments (28). These findings are supported by a forward dynamic biomechanical simulation study where increased flexor-pronator muscle mass decreased forces distributed to the ulnar collateral ligament (29). While muscle impacts on the ulnar collateral ligament cannot be directly measured and are not reflected in the calculated elbow varus torque, increased muscle mass, at the very least to the forearm musculature, may help mitigate forces on the ulnar collateral ligament, decreasing potential injury risk (30).

Growing up in different geographical regions impacts skill development and game play in baseball. How these differences influence injury risk is not well understood. The findings of this study suggest that DR-born baseball pitchers have higher elbow stress and less efficient mechanics. This increased elbow stress should be considered when developing training programs and pitching plans for professional pitchers. Coaches and player development professionals should focus on developing mechanics that limit elbow torque and focus on protecting the elbow with arm care and strength protocols. In addition, coaching staff should be especially vigilant of pitch counts during the development period as every throw compound elbow stress.

These findings necessitate future research to explore other pitching kinematic differences between DR and US baseball pitchers. Additional research should also evaluate whether increased elbow varus torque among preprofessional DR pitchers predisposes professional DR pitchers to elbow injury. Prospective longitudinal research is needed to elucidate potential pitching mechanical changes that occur between DR and US pitchers throughout their preprofessional careers. Understanding how changes in mechanics or strength training interventions affect pitching efficiency in preprofessional baseball players is also required.

There were limitations in this investigation. Kinetic calculations utilize anthropometrics estimated from cadaveric analyses, which potentially may not be representative of the participant. Unavoidable skin movement between the reflective markers and the anatomic landmarks that they are representing may decrease the precision of these biomechanical analyses. These analyses are cross-sectional and may not fully represent pitching kinematics and kinetics in this sample. While all DR and US pitchers were considered professional baseball prospects, not all participants will play professionally, decreasing the generalizability of this sample. In addition, hand velocity was used as a representative of ball velocity. Ideally, recorded ball velocity would be included in analyses.

## Conclusion

Preprofessional Dominican Republic pitchers demonstrated increased elbow varus torque and decreased hand velocity; a representative of pitch velocity when compared with United States pitchers. When controlling for confounders, Dominican Republic pitchers demonstrated even greater elbow varus torque compared with United States pitchers. Increased elbow varus torque and inefficient pitching mechanics among Dominican Republic pitchers should be considered when developing training programs and pitching plans for professional pitchers.

## Data Availability

The raw data supporting the conclusions of this article will be made available by the authors, without undue reservation.
